# Hypertension among HIV-Infected Adults Receiving Highly Active Antiretroviral Therapy (HAART) in Malaysia

**DOI:** 10.5539/gjhs.v6n2p58

**Published:** 2013-12-01

**Authors:** Nazisa Hejazi, Huang MSL, Khor Geok Lin, Lee Christopher Kwok Choong

**Affiliations:** 1 School of Health Sciences, Faculty of Health Sciences, Universiti Kebangsaan Malaysia, Bangi, Malaysia; 2 Department of Nutrition and Dietetics, Faculty of Medicine and Health Sciences, University Putra Malaysia, Serdang, Malaysia; 3 Department of Nutrition and Dietetics, School of Pharmacy and Health Sciences, International Medical University, Kuala Lumpur, Malaysia; 4 Department of General Medicine, Hospital Sungai Buloh, Sungai Buloh, Malaysia

**Keywords:** hypertension, HIV, HAART, ARV, nutrition, Malaysia

## Abstract

There are increasing researches about non-communicable disease such as elevated blood pressure among people living with HIV before and after initiation of highly active antiretroviral therapy (HAART). This cross-sectional study was designed to determine the prevalence of hypertension and associated risk factors among 340 HIV-infected patients on antiretroviral therapy at a Malaysian public hospital providing HIV-related treatment. Data on socioeconomic background, anthropometry, medical history and dietary intake of the patients were collected. Hypertension is defined as blood pressure ≥130/85 (mm Hg). Prevalence of hypertension was 45.60% (n=155) of which 86.5% of the hypertensive group were male (n=134). The results showed that increase in age (OR 1.051, 95% confidence interval (CI) 1.024-1.078), higher body mass index (OR 1.18, 95% CI 1.106-2.71), bigger waist circumference (OR 1.18, 95%CI 1.106-2.71), higher waist-hip ratio (OR 1.070, 95%CI 1.034-1.106), higher fasting plasma glucose (OR 1.332, 95% CI 0.845-2.100) and percentage energy intake from protein >15 (OR 2.519, 95%CI 1.391-4.561) were significant risk factors for hypertension (p<0.001). After adjusting for other variables, increasing age (adjusted odds ratio (aOR) 1.069 95%CI 1.016-1.124, p=0.010), being male (aOR 3.026, 95%CI 1.175-7.794, p=0.022) and higher body mass index (aOR 1.26, 95%CI 1.032-1.551, p=0.024) were independently associated with hypertension. None of the antiretroviral therapy and immunologic factors was linked to hypertension. In conclusion hypertension among PLHIV was linked to the well-known risk factors such as age, gender and body mass index. With HAART, people can live longer by making monitoring and control of some reversible factors, especially excessive weight gain for maintaining quality of life.

## 1. Introduction

There has been some evidence of an increased risk of hypertension among PLHIV (Medina-Torne, Ganesan, Barahona, & Crum-Cianflone, 2012; Nüesch et al., 2013). As a result, several studies have concentrated on identifying its underlying cause or risk factors in order to prevent or reduce its burden (Dubé et al., 2008; Diouf et al., 2012). Most of these risk factors comprise a mixture of irreversible elements such as age, gender, ethnicity and family history, and reversible lifestyle habits including smoking, diet and physical activity (Daly et al., 2012; Factor, Lo, Schoenbaum & Klein, 2013). Beside these risk factors some HIV-associated complications including renal failure and vasculopathy and/or the atherogenic effects of some anti-retroviral (ARV) agents, which result in thickening of the arterial wall thereby causing hypertension and cardiovascular diseases (Dubé et al., 2008).

Significant progress towards achieving universal access to HIV (human immunodeficiency virus) prevention, treatment and care has reduced morbidity and mortality due to the AIDS (acquired immune deficiency syndrome) dramatically (World Health Organization [WHO], 2013a). Intensifying effective HIV therapy with the combination of antiretroviral (ARV) agents as highly active antiretroviral therapy (HAART) diminishes residual levels of viral replication in body fluids or the blood to undetectable levels (WHO, 2013b). Despite all advantages of the pharmacological agents, their adverse-effects pose challenges in recent decades with new health issues. Lipodystrophy syndrome including fat maldistribution with dyslipidemia, insulin resistance and metabolic complications (Tsiodras, Mantzoros, Hammer, & Samore, 2000; Kerr et al., 2007; Hejazi, Rajikan, Choong & Sahar, 2013; Zha et al., 2013), obesity/central adiposity (Hejazi, Lee, Lin, & Choong, 2010), metabolic syndrome and diabetes mellitus (Diouf et al., 2012; Gupt et al., 2012) hypertension (Baekken, Sandvik, & Oektedalen, 2008; Diouf et al., 2012; Mateen et al., 2013) and cardiovascular diseases (Mary-Krause, Cotte, Simon, Partisani, & Costagliola, 2003; Friis-Moller et al., 2007) are some of the common side-effects of treatment with the protease inhibitors (PIs) as the third class of antiretroviral (ARV) medications. These non-communicable chronic diseases (NCCDs) with their heightened incidence have significant undesirable impact on treatment of PLHIV and their quality of life (Shenoy et al., 2013). At the same time with the HIV’s aging population, NCCDs are a growing problem among this population (Rabkin, Kruk, & El-Sadr, 2012).

There are 34.0 million [31.4 million–35.9 million] people were living with HIV at the end of 2011 globally (The Joint United Nations Program on HIV/AIDS [UNAIDS], 2013). After the first report of HIV/AIDS infection in 1986 in Malaysia, total number has increased to 81,000 people living with HIV (PLHIV) as of the end of 2011 and 14,002 of them were receiving ARV medication (Ministry of Health Malaysia [MOH Malaysia], 2012a). CVDs have been the leading cause of death for the past 40 years in the general population in Malaysia (Rampal, Rampal, Azhar, & Rahman, 2008). The National Health and Morbidity Survey (NHMS) has reported an increasing trend in the incidence of hypertension among adults aged 18 years and older (from 29.9% in year 1996 (MOH Malaysia, 1997) to 32.2% in 2006 (MOH Malaysia, 2008a) and 32.7% in 2011 (MOH Malaysia, 2012b). Notably, a prevalence rate of 43% has been reported for hypertension among Malaysian adults (age > 30 years old) in 2006 with a relative increase of 30% from that of 10 years earlier (MOH Malaysia, 2008b). Hypertension has been identified as a major risk for heath complications in this population.

Since 2006 the Malaysian government has made first line ARV medication free for all HIV infected individuals in the country. This has led to a very marked increase in the number of people receiving ARV medication. Unfortunately there has been no information about the prevalence/incidence of hypertension, related risk factors and the role of HAART among individuals on ARV medication in Malaysia. As the research question is “what is prevalence and related risk factors of hypertension among people living with HIV/AIDS (PLHIV) who are on antiretroviral therapy in Malaysia” therefore this study was carried out to determine the prevalence of hypertension and possible risk factors such as anti-retroviral therapy in a cross-sectional survey of 340 Malaysian PLHIV to alert the health professionals about the possible complications of hypertension in the near future and also to plan strategies for prevention and management of NCCDs among the PLHIV. Since cardiovascular diseases (CVDs) is the leading cause of morbidity and mortality, investigation into prevalence of hypertension which is a potential threat for cardiovascular health among PLHIV is necessary.

## 2. Methodology

### 2.1 Population and Sample

A cross-sectional study was conducted at the infectious diseases (ID) clinic of the Sungai Buloh Hospital between February and September 2009. The present study was a part of a nutritional study. The public hospital in Sungai Buloh, being the main HIV adult treatment centre in the country and is located near Kuala Lumpur in the state of Selangor, Malaysia. The study population was estimated at around two thousand subjects on antiretroviral medications from the hospital database.

A sample size of 340 adult subjects was considered adequate to determine the prevalence of the hypertension and related risk factors in these individuals. We used the sample size calculation formula which is suitable for cross-sectional studies using prevalence of health disorders in previous prevalence studies (Daniel, 1999). Since there was no available any study about nutritional status of Malaysian HIV subjects, the number of PLHIV needed for this study was calculated based on the prevalence of elevated total cholesterol (TC ≥200 mg/dL) as 67% among adult HIV-infected Thai receiving ARV medication at Bamrasnaradura Infectious Disease Institute (Pharm, 2004) which is similar to our population characteristics. In sample size calculation, the precision was for 5%, with a statistical power of 80%, a confidence interval of 95%, and with an α error of <0.05. Z Statistic for the level of confidence of 95% was 1.96 (Daniel, 1999). Base on this formula the minimum sample size was estimated as 340. Another 20% were included due to possible rejection, dropouts, missing subjects and incomplete datasets making total of 408.

Since this study comprises different ethnic groups and genders, two stage-proportional sampling methods was applied on the hospital records. The hospital database showed that 64% of the total population were Chinese (80.5% male and 19.5% female), 26% as Malay (72.7% male and 27.3% female) and 10% were Indians (83% male and 17% female) respectively. Eligible participants were Malaysian adults aged ≥18 years who had been receiving at ARV medication for at least three months and had not experienced any severe opportunistic infection during previous six months. Subjects were excluded if they had been undergoing treatment with steroids or taking oral contraceptive pill, lipid-lowering and hyperglycemic agents one month before and during ARV therapy at the time of this study.

### 2.2 Study Variables

All data about demographic, socioeconomic, HIV infection and lifestyle factors, antiretroviral therapy, dietary intake were gathered by trained interviewers and were then checked with medical records at the hospital. Anthropometric measurements were measured physically. Laboratory tests including fasting lipid profile, fasting plasma sugar, CD4 cell count and HIV RNA load were collected from medical records that were routinely collected from blood tests ordered for the same day of data collection. Of the 408 subjects, sampled from the medical records, 68 subjects did not agree to participate leaving 340 respondents in the final study.

### 2.3 Blood Pressure

Blood pressure was measured twice using a digital blood pressure machine model (General Electric, DINAMAP ProCare 120) after a minimum of 10 minute rest by the staff nurse at the ID clinic. Average of each systolic and diastolic pressure was estimated with an interval of 10 min between the first and the second measurements. Blood pressures of all respondents were measured in a sitting position on the right upper arm. In this research we categorized the subjects’ blood pressure levels based on the National Cholesterol Education Program, ATP III Third Report of the National Cholesterol Education Program [NCEP], 2002 guideline. They were divided into two groups; hypertensive subjects (with systolic BP ≥130 mm Hg and diastolic BP ≥85 mm Hg) and normotensive (systolic BP <130 mm Hg and diastolic BP <85mmHg). When a subject’s systolic or diastolic measurements were in different categories, the higher category was recorded. Subjects who were taking antihypertensive medication were assigned in hypertensive group regardless of their level of blood pressure.

### 2.4 Anthropometrics and Body Composition

Weighing was carried out without shoes and light indoor clothing using the calibrated digital Tanita weighing scale. It was recorded to the nearest 0.1kg (100g). A SECA body meter which measured up to two meters was used to measure the height of respondents. The respondents were bare footed and stood on a flat surface with their back on the tape attached to the wall. Height was recorded to the nearest 0.1(cm). Waist and hip circumference were measured to the nearest 0.1(cm) on bare skin using the SECA ® (SECA, Germany) non stretchable tape body. Respondents were told to stand still with heels together. Waist circumference (WC) in adults was obtained by measuring the distance around the smallest area below the rib cage and above umbilicus (belly button) while subjects were standing and breathing normally. Hip circumference measurements were taken at the point yielding the maximum circumference over the buttocks with the tape in a horizontal plane, touching but not compressing the skin. All anthropometric measurements of weight, height, waist and hip circumference were performed twice separately and then the average of the two readings was recorded. Waist-Hip ratio was determined by dividing mean waist circumference (cm) to mean hip circumference (cm).

The levels of body mass index (BMI) (WHO, 2000), waist circumference (WHO, 2000) and waist hip ratio (WHO, 1998) were assessed. BMI was obtained by dividing weight in Kg by height in meters squared {Weight (Kg)/height (m^2^)}. Abnormal waist circumference (abdominal obesity) was defined as WC ≥102 (cm) for males and WC ≥88 (cm) for females. Abnormal waist-hip ratio (WHR) was defined as WHR ≥0.9 for males and WHR ≥0.85 for females.

Body composition (total fat and total lean mass) was evaluated in fasting status using bioelectric impedance analyzer (BIA) BODYSTAT®1500. In accordance to the instructions in its manual, respondents were told (a) to eat or drink four hours before the test, (b) to avoid heavy exercise 12 hours before testing, (b) not to drink alcohol within 24 hours of the test, (d) to empty bladder completely prior to testing and avoid taking diuretics prior to testing unless instructed by the physician. The patients were requested to lie flat without socks and spread his/her arms and legs to avoid touching one another, after that red electrode were connected behind the right finger and right toe while black electrodes were attached to the right wrist and right ankle then the measurement was carried out. Of the 340, 2 subjects were pregnant and 4 were not able to stand up for BMI, WC, HC, WHR, body fat mass and lean mass measurements to be taken.

### 2.5 Blood Sampling and Laboratory Readings

Blood samples for the study were taken by trained medical laboratory technologists (MLTs) in the infectious disease (ID) clinic at Hospital Sungai Buloh. All laboratory studies in this research were collected from venipuncture bloods by using a size 21 gauge stainless needle punctured from right hand of fasting HIV individuals. Laboratory tests included fasting lipid profile measurements were ranked (NCEP, 2002) as high total cholesterol (TC) ≥5.17 mmol/l, high LDL- cholesterol (LDL-C) >3.36 mmol/l and low HDL-C were <1·03 mmol/l for men and <1·30 mmol/l for women. High fasting plasma glucose (FPG) was categorized as ≥6.1 mmol/l (Nüesch et al., 2013). CD4 cell count was categorized as low if CD4 count was <200 (cells/mm^3^) (Centre for Disease Control and Prevention, 1992) while detectable HIV-RNA viral load was classed (Saag et al., 1996) as viral load ≥20 copies/mL. All laboratory parameters were obtained from the computerized medical records of patients.

### 2.6 Dietary Intake

In this study the twenty-four hour recalls were carried out for two days with one on a weekday and one in the weekend. Data obtained were analyzed based on the Malaysian food database using Nutritionist Pro software (First Data Bank, 2005). If cooked dishes were not included in the database of Nutritionist Pro™ Software, Malaysian Food Composition Database (Tee et al., 2005) was used to identify recipe for each dish and then the quantitative information was entered to Nutritionist Pro software. Then the average of energy and nutrients of two days 24-hour dietary recall were used for statistical analysis. Percentage energy from carbohydrate, protein and fat were classified according to the Technical Subcommittee (TSC) on energy and macronutrients with slight modification in adopted recommendation (National Coordinating Committee on Food and Nutrition, 2005) as total carbohydrate intake should contribute up to 55%, total fat 30%, and protein 15% to total daily energy intake for the Malaysian adult population.

Total energy requirements were increased by 10 percent (WHO, 2003a) over the level of energy intake recommended for healthy non- HIV-infected persons of the same age (adults), sex, and physical activity level for asymptomatic patients in stage 1 AIDS (WHO, 2008). Dietary sodium chloride and potassium were evaluated based on WHO guideline (WHO, 2003b).

### 2.7 Statistical Analyses

All collected data using a pretested questionnaire were analyzed using software as the Statistical Packages for Social Science version 18.0 (SPSS Inc., USA). At the beginning, data cleaning was carried out to detect outliers. We found that there were no any outliers. In the second step of data analysis, the mean ±SD and percentage distribution of each variable were calculated and presented for two groups (hypertensive and normotensive) as descriptive data while significant risk factors using crude odds ratios, 95% confidence intervals (CI) and p-values were detected by univariate analysis using binary logistic regression with enter method. In the last step, multiple logistic regressions using odds ratio (adjusted OR), 95% confidence intervals (CI) and p-values was used to determine the potential risk factor for hypertension. Those variables that, in the univariate analysis, showed a p-value <0.25 for relationship/association with the hypertension were considered for multivariate model (multiple logistic regressions) by enter method. The 0.05 alpha levels were taken as the level of statistical significance for all the variables in the univariate and multivariate analyses.

### 2.8 Ethical Considerations

This study had received the approval of the Medical Ethics Committee of the Faculty of Medicine and Health Sciences, Universiti Putra Malaysia, the National Medical Research Registry of the Ministry of Health as well as the Director of the Sungai Buloh Hospital. The study procedure and objectives were clearly explained to participants using the information sheet. A written informed consent was obtained from each study subject before enrolment. Blood samples were collected by trained medical laboratory technologists (MLTs) under ethical roles of in the infectious disease (ID) clinic at Hospital Sungai Buloh Malaysia.

## 3. Results

A total of 340 subjects completed the study, giving a response rate of 83%. The study sample (n=340) was categorized in two groups: hypertensive and normotensive to detect the possible differences in mean of variables or to find out the relationship/association between variables as well potential risk factors. In the present study, hypertension was identified in 155 (45.6%) patients.

### 3.1 Socio-Demographic Factors Associated with Hypertension

Univariate analysis of demographics and socioeconomic factors found that increase in age (OR 1.05 95% CI 1.024-1.078, p=0.001) and monthly household income (OR 1.05 95% CI 1.000-1.001, p=0.002) were significant risk factors for hypertensive patients. Other potential factors, age > 40 years old (OR 1.836 95% CI 1.182-2.854, p=0.007) and household income > RM 1000 (OR 1.878 95% CI 1.213-2.909, p=0.005) also increased the risk of hypertension (Table 1) significantly. Protective factors included being female (OR 0.412 95% CI 0.235-0.722, p=0.002). Other factors, ethnicity (Malay, Chinese and Indian) and years of formal education did not contribute significantly to the presence of hypertension (p>0.05).

**Table 1:** Socio-economics and anthropometry as risk factors for hypertension

Characteristics	Normotensive Mean± SD N(%)	Hypertensive Mean ± SD N(%)	Crude OR	95.0% C.I. for OR		P*
				Lower	Upper
**Age (years)**						
Mean±SD	40.16±7.59	43.94±10.2	1.051	1.024	1.078	0.001*
<40	96 (51.9)	103 (66.5)				
≥40	89 (48.1)	52 (33.5)	1.836	1.182	2.854	0.007*
**Gender**						
Male	134 (72.4)	134 (86.5)	1.00			
Female	51 (27.6)	21 (13.5)	0.412	0.235	0.722	0.002*
**Monthly Household Income**						
Mean±SD (RM)	998.73±968.32	1445.80±1520.22	1.000	1.000	1.001	0.002*
RM <1000	94 (50.8)	55 (35.5)	1.00			
≥RM 1000	91 (49.2)	100 (64.4)	1.878	1.213	2.909	0.005*
**BMI** (**Kg/m^2^)**						
Mean±SD	21.21±3.44	23.23±3.65	1.186	1.106	1.271	0.001*
**Waist circumference (cm)**						
Mean±SD	77.57±8.90	82.82±9.40	1.066	1.039	1.094	0.001*
Abnormal	5 (2.8)	10 (6.5)				
Normal	176 (97.2)	143 (93.5)	0.406	.136	1.216	0.107
**Hip circumference (cm)**						
Mean±SD	89.67±6.84	92.38±7.28	1.057	1.023	1.091	0.001*
**WHR (ratio)**						
Mean±SD	86.48±7.10	89.58± + 6.47	1.070	1.034	1.106	0.001*
Abnormal	56 (30.9)	66 (43.1)				
Normal	125 (69.1)	87 (56.9)	0.591	0.377	0.925	0.022*
**Body Fat Mass percentage (%)**						
Mean±SD	21.68±6.87	21.16±7.28	0.990	0.960	1.020	0.506
**Body lean mass percentage (%)**						
Mean±SD	78.44±6.97	78.83±7.28	1.008	0.978	1.039	0.615
**Systolic blood pressure (mm Hg)**						
Mean±SD	114.77±9.90	145.62±12.91	1.401	1.284	1.529	0.001*
**Diastolic blood pressure (mm Hg)**						
Mean±SD	69.70±7.67	84.27±10.60	1.204	1.157	1.253	0.001*

Descriptive data are presented as mean ±SD and number (%).

* Significant association as determined by the Binary logistic regression test.

Normal WHR: Male < 0.9 and Female < 0.85.

Normal waist circumference: Male <102 (cm) and female<88 (cm).

### 3.2 Anthropometric Factors Associated with Hypertension

The findings of this study confirmed that some traditional risk factors including BMI (OR 1.186, 95%CI 1.106-1.271, p=0.001), WC (OR 1.066 95% CI 1.039-1.094, p=0.001) and HC (OR 1.570, 95% CI 1.023-1.091, p=0.001), WHR (OR 1.70, 95%CI 1.034-1.106, p=0.001) contributed significantly to the presence of hypertension. In addition the hypertensive group had higher proportion with abdominal obesity in terms of increased waist circumference and waist-hip ratio significantly (p<0.05). Body composition (body fat and lean mass percentage) was not significantly associated with hypertension (p≥0.05).

### 3.3 Metabolic Abnormalities Associated with Hypertension

Out of 340 participants only 320 had fasting lipid profile and fasting plasma glucose (FPG). Incomplete data was due to absence of laboratory results in the medical records or due to non-fasting condition at the blood was taken. The findings of metabolic parameters did not show any significant association with hypertension except for FPG (Table 2). The study found that hypertensive PLHIV had higher mean±SD of FPG than normotensives significantly (OR 1.332, CI 1.006-1.280 p=0.039). Also prevalence of raised FPS (≥6.1 mmol/l) was significantly higher among hypertensive subjects (OR 1.780, CI 1.045-3.034 p=0.034).

**Table 2:** Metabolic factors as risk factors for hypertension

Metabolic factors	Normotensive Mean±SD N(%)	Hypertensive Mean±SD N(%)	Crude OR	95.0% C.I. for OR		P*
				Lower	Upper
**Total Cholesterol (mmol/l)**						
Mean±SD	5.61±1.22	5.75±1.15	1.102	0.916	1.326	0.303
<5.17	62 (35.8)	48 (32.2)				
≥5.17	111 (64.2)	101 (67.8)	1.175	0.740	1.868	0.494
**LDL-cholesterol (mmol/l)**						
MeanSD	3.41±1.09	3.50±1.15	1.038	0.852	1.265	0.709
<3.36	85 (49.7)	68 (45.9)				
≥3.36	86 (50.3)	80 (54.1)	1.163	0.748	1.807	0.503
**HDL-cholesterol (mmol/l)**						
Mean±SD	1.15±0.37	1.15±0.34	0.990	0.536	1.827	0.974
<1.03	70 (40.5)	58 (38.9)				
≥103	103 (59.5)	91 (61.1)	1.066	0.681	1.669	0.779
**Triglycerides (mmol/l)**						
Mean±SD	2.27±1.47	2.55±1.7	1.118	0.971	1.288	0.122
<1.69	70 (40.9)	51 (34.2)	1.00			
≥1.69	101 (59.1)	98 (65.8)	1.332	0.845	1.230	0.218
**Fasting Plasma Glucose (mmol/l)**						
Mean±SD	5.46±1.87	5.95±2.19	1.332	1006	1.280	0.039*
<6.1	142 (82.6)	51 (72.7)	1.00			
≥6.1	30 (17.4)	98 (27.3)	1.780	1.045	3.034	0.034*

Descriptive data are presented as mean±SD and number (%).

* Significant association as determined by the Binary logistic regression test.

### 3.4 Dietary Intakes Associated with Hypertension

Results of dietary intake data revealed that there were no significant differences in mean energy, protein, carbohydrate, total fat, sodium, potassium, and calcium and magnesium intake between the two groups. However greater % energy from protein intake was associated with hypertension while association of higher % energy from carbohydrate intake was linked with normal blood pressure (Table 3). For finding potential factors affecting blood pressure level, a multivariate analysis of those selected factors with p-value <0.025 from univariate analyses was performed. As is evident in Table 4, after adjusting variables, increasing per year of age (adjusted OR 1.069; 95% CI 1.016-1.124; p=0.010), being male (adjusted OR 3.026; 95% CI 1.175, 7.794- p=0.022) and higher BMI (adjusted OR 1.265, 95% CI 1.032-1.551; p=0.024) contributed significantly to the risk of hypertension among the PLHIV.

**Table 3:** Dietary intake as risk factors for hypertension

**Dietary intake**	Normotensive Mean±SD N(%)	Hypertensive Mean±SD N(%)	Crude OR	95.0% C.I. for OR		P*
				Lower	Upper	
**Energy Intake (Kcal/day)**						
Mean±SD	143.27±405.84	145.08±372.59	1.000	1.000	1.001	0.670
**Protein (g)**						
Mean±SD	65.59±25.53	68.51±22.49	1.005	.996	1.014	0.270
**Carbohydrate (g)**						
Mean±SD	170.73±43.01	169.16±43.44	0.999	0.994	1.004	0.738
**Total Fat (g)**						
Mean±SD	54.13±23.50	55.47±20.88	1.003	0.993	1.012	0.581
**Sodium (g)**						
Mean±SD	1948.36± 835.56	2077.10± 835.60	1.000	1.000	1.000	0.159
**Potassium (mg)**						
Mean±SD	1345.05±490.44	1467.44±1145.49	1.000	1.000	1.001	0.224
**Calcium (mg)**						
Mean±SD	504.19 +207.970	4.92±196.05	1.000	0.999	1.001	0.585
**Magnesium (mg)**						
Mean±SD	1.576±63.018	164.64±110.68	1.001	0.998	1.003	0.470
**% Energy from dietary protein intake**						
Mean±SD	18.11±4.00	18.93±3.84	1.055	0.998	1.116	0.058
**<**15	46 (24.9)	18 (11.6)	1.00			
**≥**15	139 (75.1)	137 (88.4)	2.519	1.391	4.561	0.002*
**% Energy from dietary carbohydrate intake**						
Mean±SD	48.81+ 8.77	47.23±7.13	0.976	0.950	1.002	0.074
<55	139 (75.1)	132 (85.2)				
≥55	46 (24.9)	23 (14.8)	0.527	0.302	0.917	0.023*
**% Energy from dietary total fat intake**						
Mean±SD	32.96±7.35	33.73±6.55	1.016	0.985	1.048	0.312
<30	69 (37.5)	46 (29.7)				
≥30	115 (62.5)	109 (70.3)	1.422	0.901	2.243	0.130

Descriptive data are presented as mean±SD and number (%).

* Significant association as determined by the Binary logistic regression test.

**Table 4:** Risk of hypertension in relation to factors found significant in binary logistic regression (Odds ratios and 95% confidence intervals)

Variable	Adjusted OR§	95.0% C.I. for OR		P*
		Lower	Upper	
**Age (years)**	1.069	1.016	1.124	0.010*
<40	1.00			
≥40	0.830	0.348	1.979	0.674
**Gender**				
Female	1.00			
Male	3.026	1.175	7.794	0.022*
**Employment Status**				
Employed	1.00			
Unemployed	0.860	0.394	1.876	0.704
Self employed	0.655	0.207	2.075	0.472
**Household Income**	1.000	1.000	1.001	0.121
< RM 1000	1.00			
≥ RM 1000	0.959	0.408	2.253	0.924
**Duration of HIV Infection (years)**				
< 10	1.00			
≥10	0.567	0.244	1.318	0.187
**CD4 Cell Count (cells/mm^3^)**	1.000	0.999	1.002	0.715
**Current Supplementation**				
No	1.00			
Yes	0.625	0.331	1.181	0.148
**Length of Time on ARV(months)**	1.008	0.999	1.017	0.097
**BMI (kg/m^2^)**	1.265	1.032	1.551	0.024*
Underweight	1.00			
Normal	1.082	0.362	3.232	0.887
Overweight/Obese	0.896	0.155	5.175	0.903
**Waist circumference (cm)**	0.977	0.901	1.058	0.566
Healthy	1.00			
Unhealthy	3.261	0.577	18.427	0.181
**Waist-Hip ratio**	1.035	0.948	1.130	0.440
Healthy	1.00			
Unhealthy	1.914	0.791	4.633	0.150
**Fasting serum triglyceride (mmol/l)**	1.054	0.847	1.311	0.640
<1.69	1.00			
≥1.69	1.187	0.581	2.425	0.638
**Fasting plasma glucose (mmol/l)**	1.026	0.863	1.220	0.772
<6.1	1.00			
≥6.1	1.115	0.469	2.651	0.805
**Dietary sodium intake (mg)**	1.000	1.000	1.001	0.194
**Potassium Intake (mg)**	1.000	1.000	1.000	0.585
**% Energy from dietary carbohydrate intake**				
<55	1.00			
≥55	0.491	0.185	1.303	0.153
**% Energy from dietary protein intake**				
**<**15	1.00			
**≥**15	2.121	0.957	4.700	0.064
**% Energy from dietary total fat intake**				
**<**30	1.00			
**≥**30	1.143	0.525	2.491	0.736

* Significant association as determined by the multivariate logistic regression test.

§ Adjusted for age, gender, employment status, household income, current supplementation, duration of HIV infection, CD4 cell count, length of time on ARV, BMI, waist circumference, waist-hip ratio, fasting serum triglyceride, fasting plasma glucose, sodium, potassium, % energy from carbohydrate, % energy from protein, % energy from fat.

### 3.5 HIV and Antiretroviral Therapy Associated with Hypertension

The study also found that duration of HIV Infection (OR 0.971; p=0.302), CD4 cell count (OR 1.001; p=0.081) and viral load (OR 1.001; p=0.932) were not significantly associated with hypertension. Means ± SDs were [normotensive: 6.096±4.069 (year) *v*. hypertensive: 5.653±3.709 (year)] for duration of HIV Infection, (normotensive: 383.91±200.90 (cells/mm3) *vs*. hypertensive: 426.08±239.56 (cells/mm3)] for CD4 cell count and (normotensive: 1,884.49±16,363.26 (copies/mL) *vs*. hypertensive: 1,753.65±10,736.93 (copies/mL)] for viral load.

CD4 Cell Count <200 (cells/mm3) (normotensive: 17.3% *v*. hypertensive: 17.5; p=0.955) and viral load ≥20 (normotensive: 23.5% *v*. hypertensive: 14.2%; p=0.856) was not significant different between the two groups. Factors related to antiretroviral therapy including length of time [OR 1.006; p=0.054], changed first line of antiretroviral therapy (normotensive: 49.5% *v*. hypertensive: 51.9%; p=0.648), exposure to antiretroviral agents including Zidovudine (normotensive:53.6% *v*. hypertensive: 50.3%; p=0.554), protease inhibitor (normotensive: 45.9% *v*. hypertensive: 36.4%; p=0.927) and Stavudine (normotensive: 14.3% *v*. hypertensive:15.6%; p=0.816) were not significantly associated with hypertension.

Similarly the percentages of current nutritional supplement (vitamin and minerals) receivers (normotensive: 29.2% *v*. hypertensive: 23.2%; p=0.215) and ever drug abusers (normotensive: 18.4% *v*. hypertensive: 14.2%; p=0.301) were not significantly different between the two groups.

### 3.6 Potential Associated Factors with Hypertension

Multivariate analysis (Table 4) did not reveal that employment status, household income, current supplementation, duration of HIV infection, CD4 cell count, length of time on ARV, waist circumference, waist-hip ratio, fasting serum triglyceride, fasting plasma glucose, dietary sodium intake, potassium intake, % energy from carbohydrate intake, % energy from protein intake and % energy from fat intake did not play a significant role (p≥0.05) as cause or protective factor of elevated blood pressure.

## 4. Discussion

Hypertension as a well-known risk factor for CVDs is a health issue with a high prevalence among HIV patients. In this study 45.6% had hypertension which was higher than prevalence of hypertension in the general Malaysian population in year 2011 as 32.7% (MOH Malaysia, 2012b) and year 2006 as 43% (MOH Malaysia, 2008a).

One possible reason is due to the difference in cut-off points for definition of hypertension between the present study which used the NCEP guidelines which considered a lower cut-off point level (BP>130/85 mm Hg) than NHMS Survey (MOH Malaysia, 2012b) which defined hypertension as BP>140/90 mm Hg. Also in NHMS year 2006 most (57.6%) of the respondents were females (Rampal et al., 2008). Since the being male is a potential risk factor for hypertension and we had a higher percentage of male subjects (78%) in the present study compare to NHMS (57.6%) thus this may explain as another reason for greater percentage of hypertensive subjects in this research. Other studies (Medina-Torne et al., 2012; Factor et al., 2012; Malaza et al., 2012) also reported a lower prevalence of hypertension compare to our study due to the higher level of definition for blood pressure to be termed as hypertensive. Generally, differences in the study population characteristics, design and methodology, the type and number of variables taken as risk factors, cut-off points and references for definition of hypertension cause a variation in prevalence/incidence of hypertension.

In this study 45.6% had hypertension which was higher than prevalence of hypertension in the general Malaysian population which was 32.7% in year 2011 (MOH Malaysia, 2012b). One possible reason is due to the difference in cut-off points for definition of hypertension between the present study which used the NCEP guidelines which considered a lower cut-off point level (BP>130/85 mm Hg) and that of the National Health and Morbidity Survey (MOH Malaysia, 2012b) which defined hypertension as BP>140/90 mm Hg. Nonetheless almost half of the study population had hypertension. Other studies (Medina-Torne et al., 2012; Factor et al., 2012; Malaza et al., 2012) also reported a lower prevalence of hypertension compared to our study due to the higher level of definition for blood pressure to be termed as hypertensive. Generally, differences in the study population characteristics, design and methodology, the type and number of variables taken as risk factors, cut-off points and references for definition of hypertension cause a variation in prevalence/incidence of hypertension.

Univariate analysis found significant association between hypertension and other factors which are deemed usual and well-known causes of hypertension in HIV naïve populations. Being male gender, increase in age, higher household income, higher BMI, bigger waist circumference, higher WHR and FPS significantly contributed to the occurrence of hypertension. Vance et al. (2011), Crane and colleagues (2006) and Jung et al. (2006) also reported that older adult HIV positive were more prone to hypertension. Similarly, Crane and colleagues (2006) found that age above 40 (crude OR 1.7; 95%CI 1.0, 2.8) had increased the risk of developing elevated BP among 95 HIV patients in USA. Another study by Jung et al. (2004) among 214 German HIV positive using multivariate analysis found that increase in age (age >50) increased the risk of having hypertension (OR 7.77; 95%CI 3.41, 17.73; p<0.0001). Aging causes a loss in vessel function by stiffening of the arterial vasculature (Messerli et al., 1983). The vascular changes include the advanced reduction in visco-elastic properties of vessels, progressive atherosclerotic arterial disease, and hypertrophy/sclerosis of muscular arteries and arterioles which narrow the vessels wall and make a resistance to blood pressure and flow.

Results revealed that obesity and central adiposity, major and well-known factors for hypertension were also featured as significant risk factors. WHR increased the risk of hypertension (OR 2.45; 95%CI 1.10, 5.46; p<0.05) but BMI ≥25 (Kg/m^2^) was not a significant risk factor for hypertension (OR 1.36; CI 0.62, 2.96; p=0.45). Similar to the present study, other studies one in USA (Khalsa et al., 2004) and two in Norway (Baekken et al., 2008); Bergersen, Sandvik, Dunlop, Birkeland, & Bruun, 2003) showed that the increase in BMI was associated with hypertension. Also a study by Hejazi et al. in 2009 showed that abdominal obesity was a significant risk for increased systolic (p=0.006) and diastolic blood pressure (p=0.003) among PLHIV. It is explained by an abnormal kidney function with obesity which induces excessive renal sodium reabsorption and a hypertensive shift of blood pressure through the increased production of angiotensin II and aldosterone by obesity-induced sympathetic activation and the release of molecules by hypertrophied fat cells (Hall, 2000).

In this study male gender was a hazard factor for hypertension. The reason for difference in prevalence of hypertension between two genders is due to the sex hormones as a pathophysiologic reason (Dubey, Oparil, Imthurn, & Jackson, 2002). It is proven that female sex hormones support renal hemodynamics and prevent excessive sodium reabsorption by kidney. Thus salt sensitivity among premenopausal women is less than males (Pechère-Bertschi, & Burnier, 2004). In this study we did not find any association between most metabolic factors with hypertension except for FPG. The raised level of FPS may be due to multiple factors including the adverse-effect of antiretroviral therapy, obesity and central obesity which indirectly causes the diabetic effects such as hardened vessel wall and hypertension (Stern, Sowers, & Tuck, 2003). As important consequence, antiretroviral therapy in terms of duration, line and types of ARV agents (Zidovudine, Stavudine, protease inhibitors) and HIV infection did not contribute in hypertension occurrence. Similar to this study Medina-Torne et al. (2012) and Factor et al. (2013) did not find anti-HIV medications as a cause of hypertension. In this research metabolic parameters (except of FPS) did not play any considerable role in this disorder.

The result from dietary analysis is interesting. We found that a higher proportion of subjects with normal blood pressure had consumed more carbohydrate and less fat and protein in terms of percentage energy compared to the hypertensive group. Similarly (Straznicky, O’Callaghan, Barrington, & Louis, 1999) reported that diets with high percentage energy from carbohydrate may be accompanied by the reduction in percentage energy from fat. They concluded that short-term consumption of a high-carbohydrate resulted in lower blood pressure and fasting glucose levels and a slightly improved glucose tolerance. It is reported that glucose intolerance and increased insulin resistance may be important determinants of hypertension (Lind, Lithell, & Pollare, 1993; Sechi, & Bartoli, 1997). Additionally, the association of an increase in FPS and hypertension could merely reflect an anthropometry disorder, rather than diet rich in carbohydrates, so common among the obese. Thus nutritional intervention should be one of the priority areas in the health plan. Finally, factors such as older age, male gender and higher BMI (kg/m^2^) level are associated with a higher level of risk for the studied population in this study.

There are several limitations to the present study. Since this study investigated a cross-section of the study population, data can be specified only to the studied sample population who are predominantly male. The results cannot be applied to all PLHIV including those receiving treatment from other hospitals in the country. Since there is no follow-up about changes in hypertension status among studied population, we cannot predict the development of hypertension in the future. At the same time it should be noted that the study adopted the NCEP guidelines and included pre-hypertensive cases as hypertensive cases. Therefore comparing this group with the general population must be made with caution. Regardless of these limitations, the study provides valuable data on the importance of hypertension and causative risk among high risk HIV adults on ARV medication in Malaysia. Since it is impossible to control irreversible factors associated with hypertension, we should concentrate our efforts to assist HIV positive population adopt a healthy lifestyle by providing them with appropriate information on diet, physical activity and excessive weight gain, all of which are non-invasive ways in the prevention of cardiovascular morbidity and mortality among people living with HIV/AIDS. Investigation into the impact of hypertension on adherence to the antiretroviral medication and quality of life can be a helpful recommendation for future research in this population.

## References

[ref1] Baekken M, Os I, Sandvik L, Oektedalen O (2008). Hypertension in an urban HIV-positive population compared with the general population: influence of combination antiretroviral therapy. Journal of hypertension.

[ref2] Bergersen B. M, Sandvik L, Dunlop O, Birkeland K, Bruun J. N (2003). Prevalence of hypertension in HIV-positive patients on highly active retroviral therapy (HAART) compared with HAART-naive and HIV-negative controls: results from a Norwegian study of 721 patients. European Journal of Clinical Microbiology and Infectious Diseases.

[ref3] (1992). Centre for Disease Control and Prevention. Revised classification system for HIV infection and expanded surveillance case definition for AIDS among adolescents and adults. MMWR.

[ref4] Crane H. M, Van Rompaey S. E, Kitahata M. M (2006). Antiretroviral medications associated with elevated blood pressure among patients receiving highly active antiretroviral therapy. Aids.

[ref5] Daly R, Freemantle J, Koh G, Stradling C, van der Linde M, Taylor S (2012). April. Are traditional risk factors associated with cardiovascular events in HIV positive subjects?. HIV MEDICINE.

[ref6] Daniel W. W (1999). Biostatistics: A foundation for analysis in the health science.

[ref7] Diouf A, Cournil A, Ba-Fall K, Ngom-Guèye N. F, Eymard-Duvernay S, Ndiaye I, Sow P. S (2012). Diabetes and Hypertension among Patients Receiving Antiretroviral Treatment Since 1998 in Senegal: Prevalence and Associated Factors. ISRN AIDS, 2012.

[ref8] Dubé M. P, Lipshultz S. E, Fichtenbaum C. J, Greenberg R, Schecter A. D, Fisher S. D (2008). Effects of HIV infection and antiretroviral therapy on the heart and vasculature. Circulation.

[ref9] Dubey R. K, Oparil S, Imthurn B, Jackson E. K (2002). Sex hormones and hypertension. Cardiovasc Res.

[ref10] Factor S. H, Lo Y, Schoenbaum E, Klein R. S (2013). Incident hypertension in older women and men with or at risk for HIV infection. HIV Med.

[ref11] (2005). First Data Bank. Nutritionist ProTM, Nutrition Analysis Software.

[ref12] Friis-Moller N, Reiss P, Sabin C. A, Weber R, Monforte A. D, El-Sadr W, Lundgren J. D (2007). Class of antiretroviral drugs and the risk of myocardial infarction. New Engl J Med.

[ref13] Gupta S, Knight A. G, Losso B. Y, Ingram D. K, Keller J. N, Bruce-Keller A. J (2012). Brain injury caused by HIV protease inhibitors: Role of lipodystrophy and insulin resistance. Antiviral research.

[ref14] Hall J. E (2000). Pathophysiology of obesity hypertension. Curr Hypertens Rep.

[ref15] Hejazi N, Lee M. H. S, Lin K. G, Choong C. L. K (2010). Factors Associated with Abdominal Obesity among HIV-infected Adults on Antiretroviral Therapy in Malaysia. Global Journal of Health Science.

[ref16] Hejazi N, Rajikan R, Choong C. L. K, Sahar S (2013). Metabolic abnormalities in adult HIV infected population on antiretroviral medication in Malaysia: a cross-sectional survey. BMC public health.

[ref17] Jung O, Bickel M, Ditting T, Rickerts V, Welk T, Helm E. B, Geiger H (2004). Hypertension in HIV-1-infected patients and its impact on renal and cardiovascular integrity. Nephrology Dialysis Transplantation.

[ref18] Kerr S. J, Duncombe C, Avihingsanon A, Ananworanich J, Boyd M, Sopa B, Ruxrungtham K (2007). Dyslipidemia in an Asian population after treatment for two years with protease inhibitor-containing regimens. Journal of the International Association of Physicians in AIDS Care (JIAPAC).

[ref19] Khalsa A, Minkoff H, Cohen M, Young M, Anastos K, Greenblatt R, Levine A (2004). Hypertension in HIV-infected women: relationship to HAART in the women's interagency HIV study [Abstract]. Poster session presented at The 1st. IAS Conference on HIV Pathogenesis and Treatment: Abstract no. 512.

[ref20] Lind L, Lithell H, Pollare T (1993). Is it hyperinsulinemia or insulin resistance that is related to hypertension and other metabolic cardiovascular risk factors?. Journal of Hypertension.

[ref21] Mary-Krause M, Cotte L, Simon A, Partisani M, Costagliola D (2003). Increased risk of myocardial infarction with duration of protease inhibitor therapy in HIV-infected men. Aids.

[ref22] Mateen F. J, Kanters S, Kalyesubula R, Mukasa B, Kawuma E, Kengne A. P, Mills E. J (2013). Hypertension prevalence and Framingham risk score stratification in a large HIV-positive cohort in Uganda. Journal of hypertension.

[ref23] Malaza A, Mossong J, Bärnighausen T, Newell M. L (2012). Hypertension and obesity in adults living in a high HIV prevalence rural area in South Africa. PloS one.

[ref24] Medina-Torne S, Ganesan A, Barahona I, Crum-Cianflone N. F (2012). Hypertension is common among HIV-infected persons, but not associated with HAART. Journal of the International Association of Physicians in AIDS Care (JIAPAC).

[ref25] Messerli F, Ventura H, Glade L, Sundgaard-Riise K, Dunn F, Frohlich E (1983). Essential hypertension in the elderly: haemodynamics, intravascular volume, plasma renin activity, and circulating catecholamine levels. The lancet.

[ref26] (1997). Ministry of Health. Second National Health and Morbidity Survey 1996. NHMS II Report 1997.

[ref27] (2008a). Ministry of Health. Third National Health and Morbidity Survey 2006. NHMS III Report 2006.

[ref28] (2008b). Ministry of Health. The Clinical Practice Guideline on Management of Hypertension.

[ref29] (2012a). Ministry of Health. UNGASS Indicators Country Report / Global AIDS Response Country Progress Report (GARPR).

[ref30] (2012b). Ministry of Health. Third National Health and Morbidity Survey 2011. NHMS III Report 2011.

[ref31] (2005). National Coordinating Committee on Food and Nutrition. Recommended Nutrient Intakes for Malaysia (RNI): A report of the technical working group on nutritional guidelines.

[ref32] Nüesch R, Wang Q, Elzi L, Bernasconi E, Weber R, Cavassini M, Battegay M, Bucher H.C, the Swiss HIV Cohort Study (2013). Risk of Cardiovascular Events and Blood Pressure Control in Hypertensive HIV-Infected Patients: Swiss HIV Cohort Study (SHCS). Journal of Acquire Immune Deficiency Syndrome.

[ref33] Pechère-Bertschi A, Burnier M (2004). Female sex hormones, salt, and blood pressure regulation & ast. American journal of hypertension.

[ref34] Pharm S. P. M. I (2004). Prevalence of lipodystrophy in Thai-HIV infected patients. J Med Assoc Thai.

[ref35] Rabkin M, Kruk M. E, El-Sadr W. M (2012). HIV, aging and continuity care: strengthening health systems to support services for noncommunicable diseases in low-income countries. AIDS.

[ref36] Rampal L, Rampal S, Azhar M. Z, Rahman A. R (2008). Prevalence, awareness, treatment and control of hypertension in Malaysia: A national study of 16,440 subjects. Public Health.

[ref37] Saag M. S, Holodniy M, Kuritzkes D. R, O’Brien W. A, Coombs R, Poscher M. E, Volberding P. A (1996). HIV viral load markers in clinical practice. Nature medicine.

[ref38] Sechi L. A, Bartoli E (1997). Mechanisms of insulin resistance leading to hypertension: What can we learn from experimental models?. Journal Investigative Medicine.

[ref39] Shenoy A, Ramapuram J. T, Unnikrishan B, Achappa B, Madi D, Rao S, Mahalingam S (2013). Effect of Lipodystrophy on the Quality of Life among People Living with HIV/AIDS (PLHIV) on Highly Active Antiretroviral Therapy. Journal of the International Association of Providers of AIDS Care (JIAPAC).

[ref40] Stern N, Sowers J, Tuck M. L, LeRoith D, Olefsky JM, Taylor S I (2003). Pathophysiology of Hypertension in Diabetes. Diabetes mellitus: a fundamental and clinical text.

[ref41] Straznicky N. E, O’Callaghan C. J, Barrington V. E, Louis W. J (1999). Hypotensive effect of low-fat, high-carbohydrate diet can be independent of changes in plasma insulin concentrations. Hypertension.

[ref42] Tee E. S, Ismail M. N, Nasir M. A (1997). Nutrient composition of Malaysian food. Malaysian Food Composition Database Programme.

[ref43] (2013). The Joint United Nations Program on HIV/AIDS. Global Report 2012: UNAIDS Report on the Global AIDS Epidemic.

[ref44] (2002). Third Report of the National Cholesterol Education Program (NCEP). Expert panel on detection, evaluation, and treatment of high blood cholesterol in adults (Adult treatment panel III) final report. Circulation.

[ref45] Tsiodras S, Mantzoros C, Hammer S, Samore M (2000). Effects of protease inhibitors on hyperglycemia, hyperlipidemia, and lipodystrophy: a 5-year cohort study. Archives of internal medicine.

[ref46] Vance D. E, Mugavero M, Willig J, Raper J. L, Saag M. S (2011). Aging with HIV: a cross-sectional study of comorbidity prevalence and clinical characteristics across decades of life. Journal of the Association of Nurses in AIDS Care.

[ref47] (1998). World Health Organization. Obesity: Preventing and managing the global epidemic. Report on a WHO Consultation on Obesity, Geneva, 3-5 June 1997.

[ref48] (2000). World Health Organization. Obesity: Preventing and managing the global epidemic. Report on a WHO Consultation on Obesity.

[ref49] (2003a). World Health Organization. Nutrient Requirements for People Living with HIV/AIDS. Report of a technical consultation.

[ref50] (2003b). World Health Organization. Diet, Nutrition and the Prevention of Chronic Diseases. Joint WHO/FAO Expert Consultation. WHO Technical Report Series, no. 916.

[ref51] (2006). World Health Organization. Antiretroviral therapy for HIV infection in adults and adolescents. Recommendations for a public health approach-(Rev. 2006).

[ref52] (2013a). World Health Organization. Antiretroviral therapy. http://www.who.int/hiv/topics/treatment/art/en/index.html.

[ref53] (2013b). World Health Organization. Consolidated guidelines on the use of antiretroviral drugs for treating and preventing HIV infection.

[ref54] Zha B. S, Wan X, Zhang X, Zha W, Zhou J, Wabitsch M, Zhou H (2013). HIV Protease Inhibitors Disrupt Lipid Metabolism by Activating Endoplasmic Reticulum Stress and Inhibiting Autophagy Activity in Adipocytes. PloS one.

